# Continuous Surgical Decompression for Solitary Bone Cyst of the Jaw in a Teenage Patient

**DOI:** 10.1155/2019/9137507

**Published:** 2019-04-11

**Authors:** Lluís Brunet-Llobet, Eduard Lahor-Soler, Elias Isaack Mashala, Jaume Miranda-Rius

**Affiliations:** ^1^Division of Pediatric Dentistry, Hospital Sant Joan de Déu, University of Barcelona, Barcelona, Spain; ^2^Hospital Dentistry, Clinical Orthodontics and Periodontal Medicine Research Group, Institut de Recerca Sant Joan de Déu (IRSJD), Barcelona, Spain; ^3^Department of Odontostomatology, Faculty of Medicine and Health Sciences, University of Barcelona, Barcelona, Spain; ^4^Department of Orthopedic Surgery and Traumatology, Mount Meru Regional Hospital, Arusha, Tanzania

## Abstract

**Background:**

A solitary bone cyst or simple bone cyst is a nonneoplastic osseous lesion, with no epithelial lining, also considered as a pseudocyst. These lesions, with an intact bony wall and fluid-filled, are frequently discovered by chance in radiological studies. The etiopathogenesis has not been studied in depth, and the management remains controversial.

**Case Presentation:**

We present a clinical case of a 15-year-old boy who underwent an orthopantomography to assess the development and position of the third molars during a routine postorthodontic check-up. By chance, the X-ray identified an asymptomatic radiolucent image in the left jaw, measuring 12.0 mm × 17.8 mm and compatible with a solitary bone cyst involving teeth 35 and 36. We describe our technique for performing minimally invasive decompression of the lesion using a microperforated catheter. We describe the entire course of the follow-up, both clinical and radiological, until complete cure.

**Conclusions:**

This straightforward continuous decompression technique poses no problems for the patient, has a low risk of sequelae, and is clearly cost-effective. In view of the highly satisfactory evolution, whenever possible, we favor this minimally invasive technique for the treatment of solitary bone cysts in the jaw.

## 1. Introduction

A solitary bone cyst (SBC) is a nonneoplastic osseous lesion, with no epithelial lining, also considered as a pseudocyst [1]. Interestingly, for this idiopathic bone cavity, the term mostly used in literature is “traumatic bone cyst” or “simple bone cyst” [2]. The etiopathogenesis of mandibular SBC has not been studied in depth and remains controversial. Three main theories have been put forward to explain its origin and development: (a) an abnormality of osseous growth, (b) a degenerating tumoral process, and (c) a particular factor triggering hemorrhagic trauma [3].

SBC is generally asymptomatic. Some authors have reported symptoms such as pain and tooth sensitivity and, when the jaw is affected, possible paresthesia associated with displacement of the inferior dental canal [4, 5]. SBCs may be found in other parts of the skeleton, mainly in the long bones (90-95%) with a high prevalence in the proximal metaphyseal region of the humerus (65%) and the diaphyseal axis of the femur (25%) [5, 6]. SBCs represent only 1% of all maxillary cysts, and their incidence in the upper jawbone is infrequent: the vast majority of jaw SBCs are located in the premolar and molar regions of the body of the mandible (75%) or in the symphysis [7, 8]. Few cases have been described in the mandibular branch and/or the condyle [9]. SBCs are most frequently seen during the second decade of life, and the sex distribution is quite even [4, 10].

The most frequently recommended treatment for SBCs is surgical exploration followed by curettage of the bony walls. Surgical exploration is a diagnostic maneuver which can also be considered as therapeutic since it causes the walls of the cavity to bleed. In fact, the induction of bleeding in the cavity allows the formation of a clot which is eventually replaced by bone. Some authors have also reported cases of spontaneous resolution [9, 11].

So the treatment of SBCs remains controversial because of their healing rate and the invasiveness of the surgery. Other therapeutic options for SBCs include the use of steroid injections, autologous bone marrow injection, open curettage and bone grafting, various methods of decompression, the use of calcium phosphate and calcium sulfate, and cannulated screws [12–15]. Traditionally, the gold standard procedure has been curettage and bone grafting, but the cure rates reported have been as low as 40% [16, 17]. In long bones like the humerus, SBC decompression can be achieved using needles or implants, such as intramedullary nails, Kirschner wires, and cannulated screws or pins. This decompression technique using intramedullary nails achieves cure rates of almost 70%, with recurrence rates of <10% [17–19]. In summary, the selective procedures for the treatment of this lesion range from the simple exploration of the cavity, including fenestration and aspiration, to condyle osteotomy in cases in which the condyle is affected.

This case report describes a presentation of a typical SBC located in the body of the mandible. The main aim is to report the favorable outcome of a minimally invasive procedure using a simple cannulation. Rapid and satisfactory recovery was achieved through continuous decompression treatment.

## 2. Case Presentation

**Figure 1:**
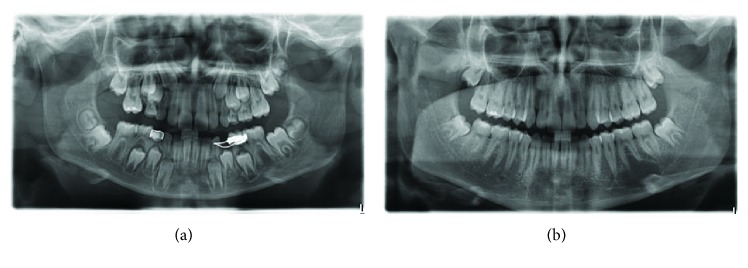
(a) Orthopantomography in the phase of mixed dentition. A normal rate of tooth eruption, without any radiolucent lesion, is seen in the area of the third quadrant. (b) Follow-up orthopantomography of the third molars after orthodontic treatment. The radiolucent lesion affecting the areas of 35, 36, and 37 is diagnosed.

**Figure 2:**
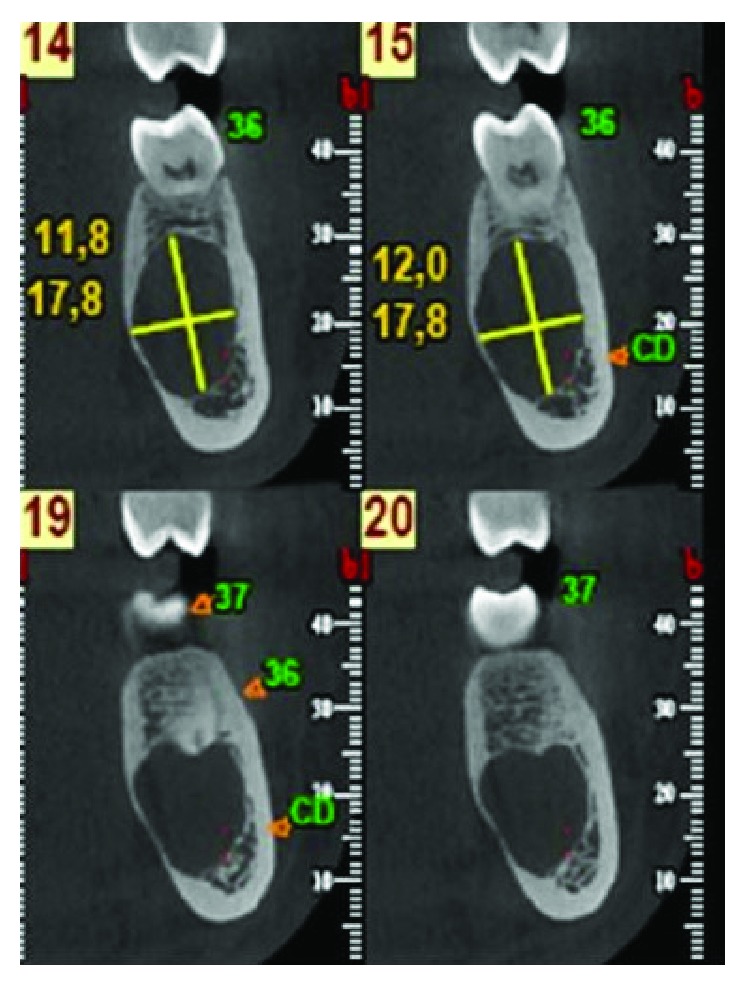
Cone beam computerized tomography. A well­defined unilocular radiolucent lesion of 12.0 × 17.8 mm is observed, with preservation of both the root apex and the cortical bone. However, a marked thinning of the lingual cortical mandibular plate is seen.

The 15-year-old patient had no pathological history of interest. By chance, during a routine postorthodontic visit, an orthopantomography of the third molars identified a radiolucent image encompassing the periapical area of teeth 35 and 36 (Figures 1(a) and 1(b)). Cone beam computed tomography (CBCT) confirmed the presence of a well-defined radiolucent unilocular lesion, with preservation of the root apices of the teeth involved and the cortical bone. However, the lingual cortical plate was observed to be extremely thin, due to the compressive effect of the pseudocyst itself (Figure 2).

**Figure 3:**
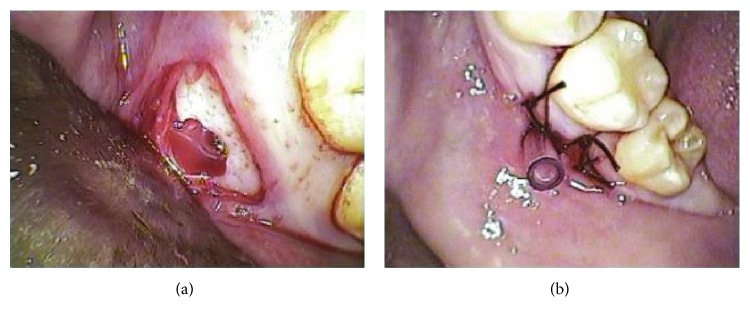
(a) Clinical image of the mucoperiosteal flap for accessing the buccal cortical plate and for performing trepanation to allow drainage of an amber-colored serohematic fluid. (b) Cannulation of the lesion via a microperforated Nelaton catheter.

Minimally invasive surgery after minor flap reflection was planned in order to access the buccal cortical plate of the lower jaw. Using digital radiography, a favorable interradicular point was located to avoid damaging the roots of the molar. Trepanation was performed with a bone surgery burst diameter, reaching a space from which amber-colored serohematic fluid, a characteristic of these lesions, was subsequently drained. Finally, the bone cavity was cannulated through the end (3.5 cm) of a Nelaton-type catheter leaving a 1 cm section exposed which was fixed with silk 3/0 (Figures 3(a) and 3(b)). Previously, multiple perforations had been made along the most distal part of the catheter so as to facilitate drainage and minimize the risk of obstruction.

Unfortunately, histological analysis was not possible due to insufficient quantity of the sample. As a result, a presumptive diagnosis of SBC was established on the basis of the patient's medical history, radiological images, the lack of clinical symptomatology, and the fluid drained during the surgery.

Periodical controls were performed every 15 days including radiographic inspection of the lesion and irrigation with physiological serum so as to ensure the permeability of the drainage holes. The vitality of teeth 35 and 36 was also monitored.

**Figure 4:**
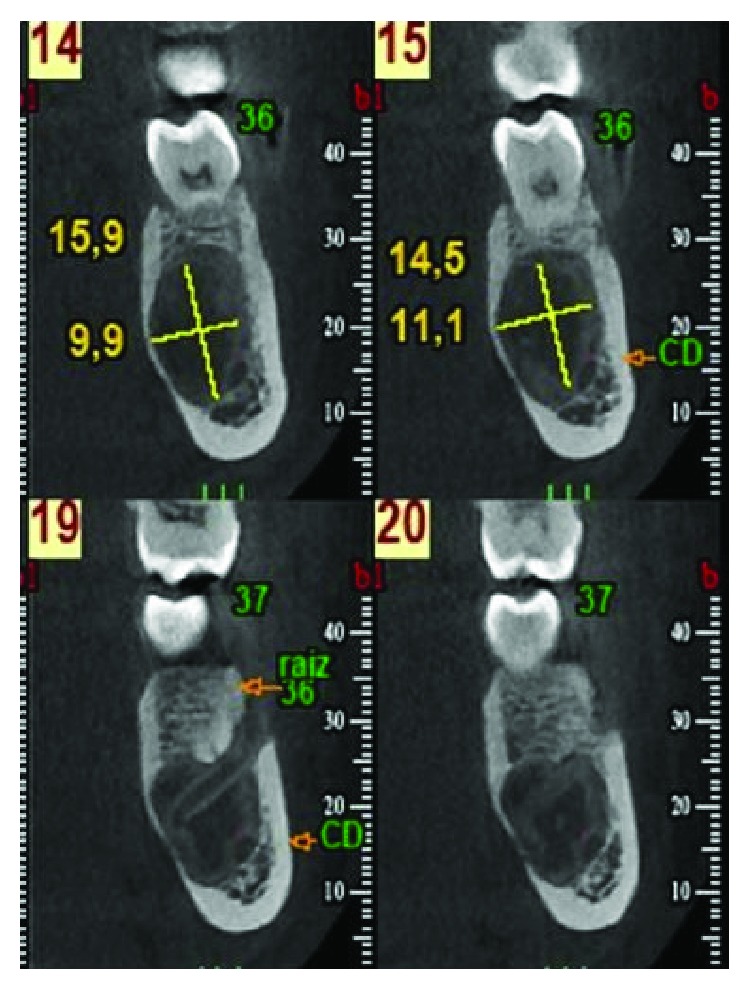
Cone beam computerized tomography 2 months after surgery. A slight reduction in lesion size (11.1 × 15.9 mm) is seen. Note the incipient recovery of the cancellous bones in the radiolucent image. The drainage catheter placed in the area of the lesion can also be seen.

**Figure 5:**
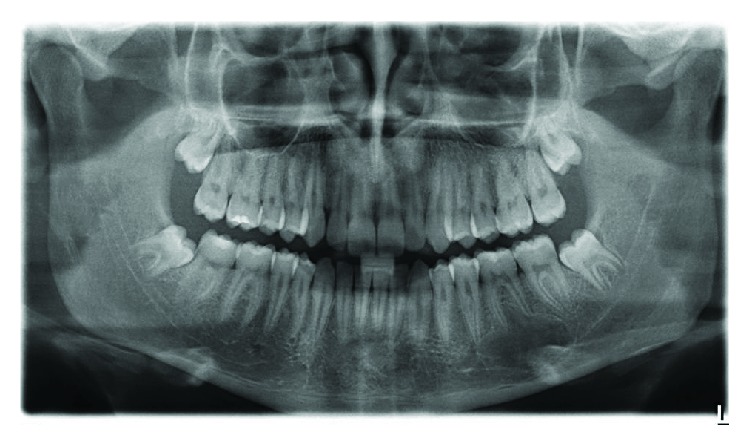
Control orthopantomography 5 months after surgery, showing a significant improvement in bone density at the level of the cavity.

**Figure 6:**
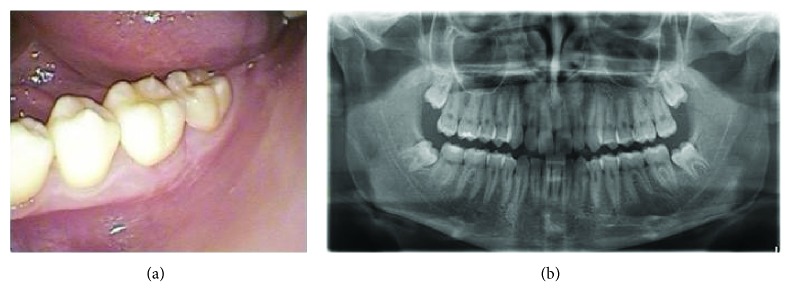
(a) Clinical image: excellent scarring of the area where the drainage catheter was placed. (b) Control orthopantomography 9 months after surgery. Significant reductions are seen in the size of the lesion and in the presence of cancellous bone.

**Figure 7:**
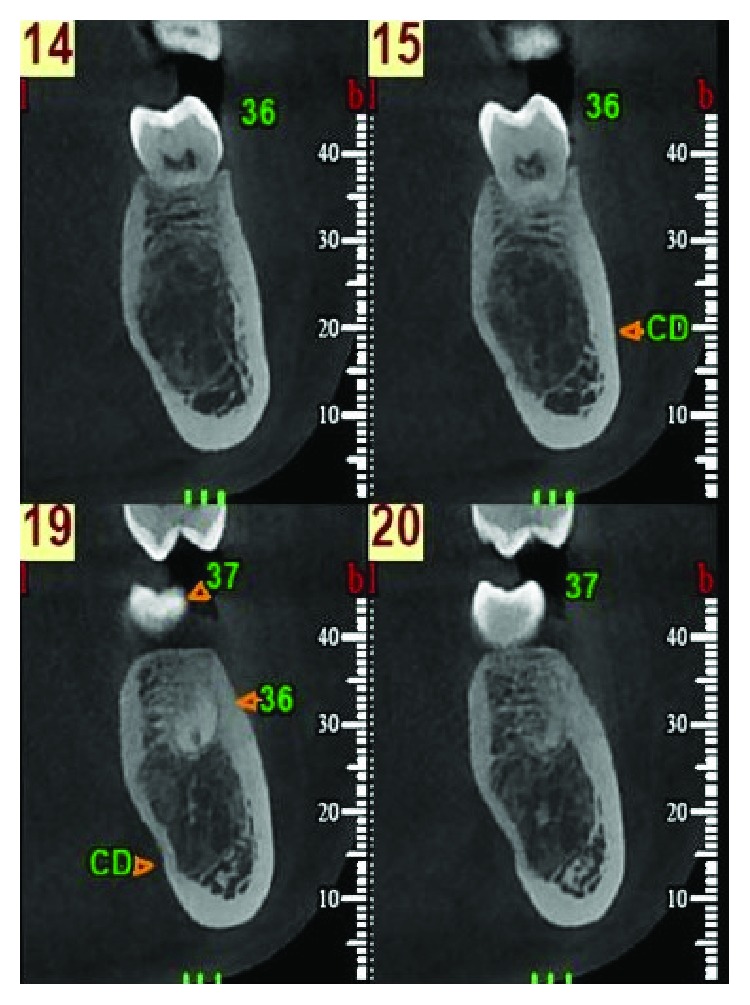
Cone beam computerized tomography within 18 months of canalization. Complete replacement of the radiolucent image is observed, with a density compatible with normal bone tissue in the process of calcification. The lingual cortical plate adjacent to the lesion in the area of teeth 35-37 presents normal thickness.

**Figure 8:**
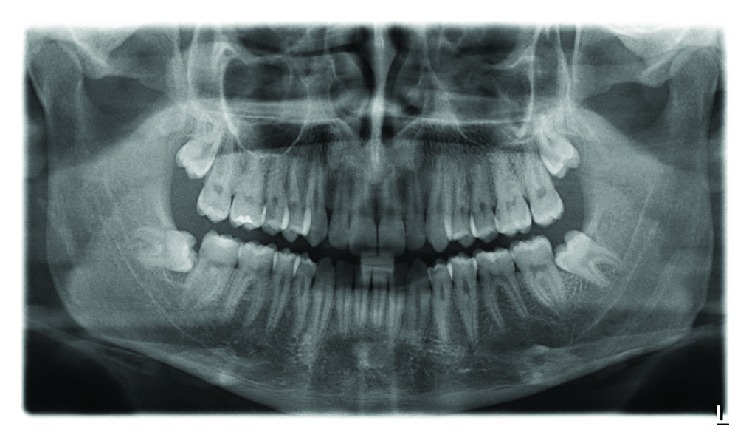
Control orthopantomography 24 months after surgery. Note the complete recovery of the lesion.

After two months, a new CBCT was requested to inspect the area of the lesion, which indicated the incipient recovery of cancellous bone in the radiolucent image (Figure 4). Five months after treatment, a control orthopantomography indicated improved bone density at the site of the lesion (Figure 5). At nine months, a new orthopantomography showed a significant reduction in the size of the lesion and a significant increase in the cancellous bone inside (Figures 6(a) and 6(b)). Surprisingly, at 18 and 24 months after the surgical cannulation, the pathological image presented a bone density compatible with normal bone tissue in the process of calcification, and indeed, it was difficult to define the original lesion (Figures 7 and 8). In addition, the thickness of the lingual cortical plate adjacent to the lesion in the area of teeth 35-37 was normal (Figure 7).

## 3. Discussion and Conclusions

SBCs of the jaw are often asymptomatic, with no inflammation and no effect on function; often, the vitality of adjacent teeth is also unaffected. These lesions, with an intact bony wall and fluid-filled, are frequently discovered by chance in radiological studies [7, 20, 21]. In our case, the SBC was a chance finding during an orthopantomography carried out as part of an annual routine visit. However, some patients report pain, inflammation, and/or dental sensitivity [22]. The presence of fistulas, root reabsorption, paresthesia, and/or pathological fractures associated with these idiopathic bone cavities is scarce [23]. SBCs rarely cause complications, but the possibility of pathological fractures in extensive lesions should not be ruled out [24]. When SBCs of the jaw are associated with bone cement dysplasia, cementoma, odontoma, or mesodermal tumor, patients may present pain or inflammation [6, 24–26].

On radiography, many solitary cysts appear as radiolucent lesions with margins that may be irregular and partially sclerotic mixed with cancellous bone, but they are well defined and have a normal appearance [7, 21, 22, 27].

The main characteristic of SBCs is scalloping when they extend towards the dental roots; this scalloping is also described in edentulous areas [9, 23, 28]. Another radiographic feature of SBCs is the broad extension of the lesion without causing bone expansion; the cortical bone tends to be thinned due to intraosseous erosion. This characteristic can be observed in the CT images of the case presented here with lingual cortical thinning, no displacement or reabsorption of adjacent teeth, and preservation of the lamina dura.

The etiology and pathogenesis of these bone cavities are not well established. Trauma can be an important factor in their development, although its mode, intensity, frequency, and pathogenesis must be determined before any firm conclusions can be reached [9]. In the case presented here, the patient did not recall any major trauma; however, the fact that he wore braces might be included as a possible cause of low-intensity but constant traumatism over time [29]. Since the material available for histological study is often scarce, it may be difficult to obtain sufficient evidence for a definitive diagnosis [5]. However, almost all histological findings reveal fibrous and normal bone tissue. Generally, there is no evidence of an epithelial lining, although in some cases the cavity may be lined by a thin fibrous membrane. The lesion may exhibit areas of vascularity, fibrin, erythrocytes, and occasional giant cells adjacent to the bone surface [11, 30]. Histological analysis could not be performed in our patient, but after such a favorable evolution, we might also consider a diagnosis *ex juvantibus*: that is, we might infer the cause of the disease from the response observed to the treatment.

Peñarrocha-Diago et al. [7] agreed that teeth with apexes involved in the lesion should not undergo endodontic treatment, since prognosis is favorable and normal healing occurs without any further complications. In the same study, these authors argue against the curettage of the roof or floor of the cavity, so as to preserve the vitality of adjacent teeth [7]; in contrast, others suggest that devitalization of teeth within the lesion site is a factor that may affect solitary bone cyst healing [26]. Cases of secondary pathological fractures have been described following SBCs [17]. Fortunately, at the time of our radiological diagnosis, our patient's lingual cortical plate, though notably thinned, was still conserved.

The decompression technique with needles has been used to treat SBCs in children's long bones [18]. In children's jaw bones, the use of the Nelaton catheter incorporated into a Hawley plate has been described for reducing the size of major cystic lesions [31]. Nonetheless, the insertion of a removable appliance with a catheter can be difficult for the patient, and the entry path often tends to reepithelialize, obliging the repetition of the defenestration through the gingival mucosa. We stress the benefits of our approach for patient comfort, since there is no need for a removable device or for any handling of the catheter inserted after the minimally invasive surgery. The main advantages of the Nelaton catheter are the fact that it is made of nontoxic, nonirritant medical transparent polyvinyl chloride (P.V.C.) material and that it has a smooth surface with two lateral eyes for efficient drainage and the distal end coned for nontraumatic introduction. Our patient was treated with a continuous decompression procedure for two months using a Nelaton-type cannula with various lateral orifices. This also allowed the removal of the cavity liquid content and favored bone regeneration at the site of the lesion. At all times, up until the complete regeneration of the lesion, the vitality of the teeth involved in or adjacent to the SBC remained normal and unchanged. In locations other than the lower jaw, some authors recommend the use of hydroxyapatite pins [17]. However, in the jawbones and in certain locations that are difficult to access, it is easier to carry out the decompression with flexible cannulas rather than with needles or implants. In fact, in the case we present here, not even infiltrative anesthesia was required to withdraw the cannula.

In summary, this continuous decompression technique is straightforward, causes no inconvenience to the patient, and has a low risk of sequelae and a clearly favorable cost-benefit relationship. In view of the highly satisfactory evolution observed here, whenever possible, we propose this type of minimally invasive technique for the treatment of SBCs in the jawbones.

## Abbreviations

SBC: Solitary bone cyst
CBCT: Cone beam computed tomography.
